# The Single Remedy

**Published:** 1882-07

**Authors:** P. P. Wells

**Affiliations:** Brooklyn, N. Y.


### "THE SINGLE REMEDY."

P. P. Wells, M. D., Brooklyn, N. Y.

In the May number of the *North American Journal of Homœopathy* is a paper which purports to have been written by Dr. W. S. Searle, and to have been read by him "before the Medico-Chirurgical Society of New York." It is notable for its utterances, with some of which it is our purpose now to deal, but more for the vivid exhibition of some of the characteristics of the writer, which can be of little consequence to any one but himself. In so far as the objective of this paper was to exhibit himself, it must be admitted the writer has achieved a remarkable success. Beyond this it is not very apparent that he had a motive which calls for notice. It is sometimes the object of a writer to instruct others who know less than himself. This is always worthy. But if this were the objective of the writer of this paper, judged by its contents, we do not immediately see where he is to find his pupils. For the sake of these and the public, it is to be hoped, when found, the class will be small. The paper reads throughout just like the utterances of a tyro, who in his strivings to comprehend the first general principle of the philosophy he professes faith in, finds its scope beyond his grasp, and who has remained in the agony of these efforts; never having been able to get beyond them, and to have become so wholly absorbed by them as to have remained in utter ignorance of all beyond, and in entire incapacity to comprehend the initial object of his strivings, *i. e.*, as to all that makes up the philosophy of Homœopathy, he has remained in helpless, hopeless infancy, from the imbecility of which this paper gives little hope of his ever emerging.

Indeed, the confirmed infancy and imbecility of his practical life seems fully confirmed by his utterance on the specific relationship of *Bell.* to its characteristic form of uterine hemorrhage; a relationship than which there is, we believe, no other in the range of practical therapeutics more certainly established by clinical experiences. This is well known, and has long been well known, to those who have so far emerged from the ignorance of infancy and tyroism as to have attained only a very moderate share of knowledge of the materia medica. And yet this is the way he speaks of this fact, and those who know it to be a fact: "I have seen such dabblers gravely discussing whether to give Belladonna in a case of flooding, on the basis that the clots were dark and the liquid blood bright red." And he says: "These men were not tyros, either." Presumably they were not. And then he cries, in an air of conscious triumph: "Where was their pathology, and their common sense?" We don't know where their "pathology" was, but we have a pleasing evidence that their "common sense" was employed in loyally obeying God's law of healing, where, if he is ever found imitating their example, he may undoubtedly find it. You see this man very likely means well, but the trouble with him is, he don't know. Then why spend time and paper on this essay? Is it to enlighten its author? No. That may be safely accepted as impossible. He is not enlightened by the little of truth he has discerned. Then why give him more? It is the *acceptance* of the truth which gives light. It is putting one's self under the guidance of this light that brings wisdom.

The very first utterance of the paper would seem to show that the writer had really some inkling of the truth of the matter he was about to discuss. This is it:

"The dogma [of the single remedy] * * * is a legitimate outgrowth and a logical deduction from the basic law of the system established by Hahnemann." This is true and well put. Being true, then another logical deduction follows—that for all who profess faith in, and who pretend to practice that system, this matter of the "single remedy" is settled, *i. e.*, for all who are amenable to law and logic. It will not be our fault if before we are through with this paper we find evidence that its author is not of this number.

We have intimated that our author found difficulty in his efforts to comprehend the initial principle in Homœopathy, and that evidence of this fact pervades his paper. We understand this principle to be the law of cure—God's law, and not man's. We understand Dr. S. is supposed to have accepted this law. And that he and all who accept it find its briefest expression in the phrase, "*Like cures like.*" Now the difficulty with the doctor is, he seems to be wholly without definite perception of the "*like*" which cures. He talks of this law as the "God-given and God-established truth," but it is clear from his statements that he has no idea of the like which this law requires. It requires one fact or class of facts to be like another fact or class of facts. Before the like can be known it must be compared with the fact or facts to be cured. Before the comparison, both facts or classes must be known. Now, in his second statement of the necessities "to a genuine homœopathic prescription," he gives first place to "physiological and pathological knowledge," "which we are to apply to them [diseases] as remedies." This is as queer a statement as any we remember to have met in writings, even the most muddy. How these sciences are to be so applied, "as remedies," the one being the science of life in health, the other of life sick, is not very apparent. It is the science of therapeutics which gives us the knowledge of how to cure. And this avails itself of materia medica for its means by which cures are to be effected.

Physiology is useful to the prescriber not as a "remedy," but as a start point from which to measure the aberrations produced by the impact of the morbid cause from the healthy balance of the actions of the life-forces. Pathology teaches the nature of these aberrations, and is in no sense and never a "remedy" for them. If it were the facts of this nature to which, in our clinical endeavors, we were to find a simillimum, the case would be bad for the patient at least, for, before this could be ascertained, the inspection of his internal condition would be called for—his pathology. This is it of which we hear so much, and of which our author so often and so fondly speaks. Before we can possibly know this he must be dissected, and even then we may be left in uncertainty, for this is not always revealed in change of tissues. Therefore, this factor in clinical duties is always more or less a matter of guessing, at the best. And then, if the simillimum the law requires us to find in materia medica be one of condition (the pathological idea), then before this can be known, the prover will have to be dissected, too, and then there will still be the same uncertainty as in the dissection of the patient, provings not being often carried to the extent of change of tissue or of visible change of condition. God has neither "given" nor "established" any so foolish law of cure as this. He has not so mocked the suffering sick. And yet we are constantly hearing of the necessity of incorporating this pathology, this *supposed* internal condition into our therapeutics, and those who so talk, think themselves wise. They have only taken the apostle's first step toward wisdom; they have only become fools. The like which cures is a like which can be known. It is found alone in facts in the materia medica which are most like the facts of the totality of the symptoms. Many of those who, like our author, are not quite capable of coping with and managing this totality in their practice, as we shall by and by find he is not, seek escape from exposure of this imbecility in sneers at this fundamental factor in all homœopathic prescribing. This they are all capable of, and it must be admitted they take an easier course than that of finding their simillimum.

Our author, as others like him have often done before, having dishonored a law he acknowledges to be "God-given and God-established," by endeavors to incorporate into clinical duties elements wholly foreign to its requirements, and having thus, as could not have been otherwise, met often clinical disappointments, they turn for defense, before their consciousness of failure, to abuse of our materia medica, and in the enjoyment of this luxury they seem to find much food for their self-complacency. To abuse a great work, why, of course he who does this must, in his own eyes, be greater, and this must be a comfort to him, and we know of no other excuse for this paltry work. We have heard much of this in our day, and chiefly from those who really knew very little of that which they have so freely disparaged. We have found it, all and always, a cheap kind of talk by a very cheap kind of men. The very mean nature of this talk is declared by the fact that of all who have thus decried the character and value of this precious fruit of other men's labors, none of those who have so loudly clamored for a winnowing of the chaff from the wheat have made the first effort to show us which is the chaff or which the wheat, and have made no personal effort for the winnowing. They have only insisted upon it that there *is* chaff and a good deal of it. By what criterion they have decided the chaff to be chaff we have not been told, but only that the chaff is chaff and it should come out. Cheap! But our author is more frank than the multitude of those with whom he thus fraternizes. He acknowledges, changing the figure, that he cannot tell the "weeds" from the wheat, they look so just alike. And more than this, he says, page 615: "The worst of it is, no man of us can certainly tell the grain from the weeds." Then it is a fair question, how does he or they know that these so-called weeds are really weeds and not wheat? They look so exactly alike that no man can tell which is which, certainly one is tempted to believe, after this confession, that he or they together *know* very little or even nothing at all about it.* And yet this inability to see the difference between the true and the false was no bar to our author's declaration that "it is probable one-third of the symptoms of *Allen's Encyclopædia* have no business whatever where they stand." How probable? How does he know this when he so frankly confesses his inability to

---

* He gives an instance of his having discovered one of these ridiculed weeds (ridiculed by one who claimed authority for his sayings because, forsooth, he had been "a teacher of materia medica in one of our colleges" and, therefore, he thought he "ought to know,") to be wheat after all. And more than this, the discovery has helped him to make cures which, but for this weed which was wheat after all, he might not have made. [Page 616.] Is it not just possible that the other so-called weeds may be wheat, and only need men with knowledge and brains enough to find this out? It may be better adapted to the endowments of these carpers to cry weeds, though.

discriminate the false from the true? It is pretty clear he had forgotten his confession when he said this. The confession no doubt is true. He changes his figure and says: "But still the unpleasant truth remains and forces itself upon us: we are far from having a materia medica pura. Why this should be so, the sources of the impurity of this boastedly limpid stream, we cannot stop to inquire." No wonder he was in a hurry to go on from a further consideration of the troublesome subject, having discovered and acknowledged his inability to distinguish, in materia medica, the true from the false, or, to continue his last metaphor, the water from the mud. Don't know. That is what is the matter with the whole tribe of carpers at our materia medica.

Indeed, "don't know" seems to be the "characteristic symptom" which runs through this paper. It appears again, on page 618, thus: "A successful prescription may be termed accidental when the physician makes it upon a symptomatic basis only, in accordance with the law of cure. It is properly so named because the vaunted totality of symptoms is not an infallible, nor always even a safe,, therapeutic guide." How does he know this? This paper seems to give abundant evidence that he has never tried it. For example the following extracts show only too clearly that he has no practical knowledge of what this "totality" really is, and gives convincing proof of his utter inability to obtain this practically when needed, and so, to him and his like it can be neither "infallible" nor "safe," not being by them found. Again, on the same page:

"Take, for illustration, a case of vomiting. The symptoms, both subjective and objective, may be exactly the same whether the disturbance be due to uræmia or to simple gastric or bilious disorder. Would any one worthy of the name of physician prescribe on a symptomatic basis only?"

We can only answer to this, we are perfectly sure no one worthy of the name of *homœopathic* physician can prescribe for this on any other basis. He evidently has no conception of the extent of this basis. This kind of physician has no other basis for a prescription, and this for the simple reason there *is no other basis for a prescription founded on law.* And again, on the same page:

"Those protean disorders, diarrhœa and headache* and a vast

---

* "Diarrhœa and headache," if we may believe a statement made, as we were told, many years ago by the writer of this paper, to a wealthy merchant and manufacturer of our city, would seem to have been very considerable and variety of other maladies might furnish us with equally opposite [presumably he wrote apposite] illustrations of the truth that something more than the mere totality of the symptoms is requisite to the genuine homœopathic prescription."

And further, on the same page, he tells us this "something more" is "pathological investigation." And then, on the same page, declares this pathology idea to be "imperfect and misleading." Imperfect and misleading because only guessed at.

The above extracts are conclusive as to the muddled ideas of our author. In uræmic poisoning and gastric disorder he sees only the vomiting—one symptom—and this is the same in each, so he says, though this may be doubted, and proceeds to his conclusion from this that "something more" is needed in finding a relief for this than a proper compliance with the "God-given" law, and he has found this "something more" to be "pathological investigation!" Suppose he had tried the guidance of the *totality* of the symptoms instead of going off after this one, in violation of the requirements of the "God-given" law, it is quite possible he would have found little need of any "thing more" than the simillimum of these for his cure. Then, how does he propose to prosecute this "pathological" investigation? By what means outside of this "totality" can such an "investigation" be prosecuted? These symptoms tell him all he can ever know of the "pathology" of his case. *They are its pathology*, and besides these and what they teach, all of so-called pathology, meaning by this some internal condition not disclosed by this totality, is wholly a matter of pure conjecture, *i. e.*, guessing. Now, how it is needful or possible to supplement profitably the teachings of this totality, our only guides as well to our knowledge of any internal condition we can know, as to the discovery of the simillimum which cures, is beyond the power of an intelligent mind to understand. This vomiting is to him his whole case. So of his other "opposite illustrations." They are but one element of a sick condition, his "diarrhœa" and "headache." He may be assured that those who loyally and intelligently obey the law in the matter of its required guidance to a knowledge of a required simillimum, are not subject to the embarrassment he seems troublesome bugbears in his professional life. Indeed, he had found them so great he was said by this merchant to have assured him that "Homœopathy would not cure these diseases!" Suppose he had found the "totality of the symptoms" of his cases, and attempted their cure on this basis!

to have endured in dealing with the elements of a sick condition which he names as requiring "something more."

On this same 618th page the doctor says: "But when obliged to depend on the totality alone, we really 'go it blind.' * * * we prescribe in ignorance of the real nature of the disease," etc. This is only a further evidence of his utter ignorance, practically, of what this totality really is. It gives the only light as to this nature it is possible for man to have. It is a queer "going it blind" when in this clearest possible therapeutic light. Bad men have been said to prefer darkness to light, but truly he must be grossly ignorant who mistakes light for darkness. It is not, therefore, surprising when we find him, after attempting a description of a success of a true homœopathic prescription, saying, "Now, are not such instances rare in your experiences?" *i. e.*, of the members of the Medico-Chirurgical Society, "I grieve to say they are in mine." Of course they are. How could it be otherwise in the experience of one who apparently knows nothing of the homœopathic law, of its spirit, or its corollaries, and in his clinical endeavors, presumably, sets them all at naught, as he advocates others doing in the paper under our hand. It is rather mean, though, for this reason, to turn on our law as inadequate to our therapeutic needs, its corrollaries as insufficient guides, and our materia medica as a muddy stream, simply because he is ignorant of the law and its corrollaries, and in materia medica he cannot distinguish the wheat from the chaff.

We shall consider but one point more of this paper, that of its title. The single remedy, which in the beginning he says is "the legitimate outgrowth and logical deduction" of a "God-given law." This is true. How is it, then, that he talks in this wise? * * * "The conscientious homœopath finds himself unable to fully satisfy his mind as to a single remedy. * * * Two, three, four occur to his mind, but he is unable to distinguish conclusively between them. What shall he do?" We should advise him very earnestly if he does not know, to pursue his study of the symptoms of his case and his materia medica till he does, and so decide his case according to law, and thus secure the greatest possible safety to his patient. Is this the doctor's idea? Not at all. He advises giving all four of these uncertainties *at the same time*,* and says of the prescriber, "*he is criminal if he does not.*" [Italics the doctor's.]

* If we are not mistaken we have read or heard, "*two* wrongs cannot make *one* right," but, truly, this is the first time we ever heard *four* uncertainties recommended as equal to making *one* certainty.

And this is the outcome of the doctor's discussion of this important therapeutic principle, which he says is the legitimate outcome of God's law. It is not only advice to others to disregard the legitimate and logical outgrowth of this law, but even to declare him "*criminal*" who will not do this. That this is his best idea of therapeutic duty will hardly surprise any reader of his paper, in which he can hardly fail to find so great ignorance of all belonging to Homœopathy, and so frequent indications of setting aside its most important practical rules. In view of the paper as a whole, it can be a surprise to no one when he declares that the natural results of a true homœopathic prescription have been rare in his experience. It could not have been otherwise. For if his practice has been in accord with this paper, it has been as far removed from all which belongs to Homœopathy as is Mohammed's Koran.

Then, it may be asked again, why waste time and paper on so great a folly as this paper proves itself to be? The reply is, even a great folly acquires a certain importance by appearance *in print*, and so, as in case of this paper, it may, by this fact, become a decoy to the beginner, leading him altogether astray from truth and success. He found these utterances in a book, so, therefore, he accepts them; being himself but a tyro and not yet having learned the truth, he receives such confident utterances of the false, chiefly because they come to him with the indorsement of the covers of a book. And he does this all the more readily when the false (as in this paper) is presented to him with air and pretense *quasi scientific*. There is very much of this afloat in our literature, and it is more or less operative in demoralizing the young and inexperienced, and the more when, as is often the case, the *pseudo scientific* is accompanied by so charming a companion as is a *pseudo liberality*. This paper has been written, not because of any supposed particular merit or demerit in that which it has discussed, but from a conviction of duty—duty so far as the writer may be able to protect the coming generation from the bad influence of that class of writing of which this paper of Dr. S.'s is a very unimportant member. If *God* has "given" and "established" a law of cure, as our author affirms, then let no man or men intrude into its administration elements not included in it and its legitimate corollaries, on any false pretense that so doing is either "scientific," or "liberal." The beginner may be assured that by whomsoever recommended, or by whomsoever practiced, this proceeding is, *quo ad hoc*, a virtual repeal of God's law.

It is not conceded to any being less wise than the Omniscient that he can rightfully or profitably do this. If such an one does this and then finds the natural results of a " genuine homœopathic prescription " " rare " in his "experience," let him not "grieve," but repent and turn from his sins to obedience to God's law and we venture to assure him, as he mends his ways his practical experiences will be found less a grief to him.

